# Supplementary material for: PITX1 protein interacts with ZCCHC10 to regulate hTERT mRNA transcription
Source: PLoS One. 2019 Aug 12;14(8):e0217605. doi: 10.1371/journal.pone.0217605 (PMC6690549; doi:10.1371/journal.pone.0217605)
Supplement: S1 Fig — ‘p’ refers to the short arm of each chromosome, and ‘q’ refers to the long arm. Numbers indicate chromosome number. Red arrow shows 6 genes coding on 5q. (DOCX) [file pone.0217605.s001.docx]

**PITX1 protein interacts with ZCCHC10 to regulate *hTERT* mRNA transcription**

Takahito Ohira^1,4^, Hirotada Kojima^2^, Yuko Kuroda^1^, Sayaka Aoki^1^, Daigo Inaoka^1^, Mitsuhiko Osaki^3,4^, Hideki Wanibuchi^5^, Futoshi Okada^3,4^, Mitsuo Oshimura^4^, Hiroyuki Kugoh^1,4*^

^1^ Department of Biomedical Science, Institute of Regenerative Medicine and Biofunction, Graduate School of Medical Science, Tottori University, Yonago, Tottori, Japan

^2^ Department of Immunology, Graduate School of Medicine, Osaka City University, Asahi-machi, Abeno-ku, Osaka, Japan

^3^ Division of Pathological Biochemistry, School of Life Science, Faculty of Medicine, Tottori University, Yonago, Tottori, Japan

^4^ Chromosome Engineering Research Center, Tottori University, Yonago, Tottori, Japan

^5^ Department of Molecular Pathology, Graduate School of Medicine, Osaka City University, Asahi-machi, Abeno-ku, Osaka, Japan

*Corresponding author

E-mail : [kugoh@med.tottori-u.ac.jp](mailto:kugoh@med.tottori-u.ac.jp)


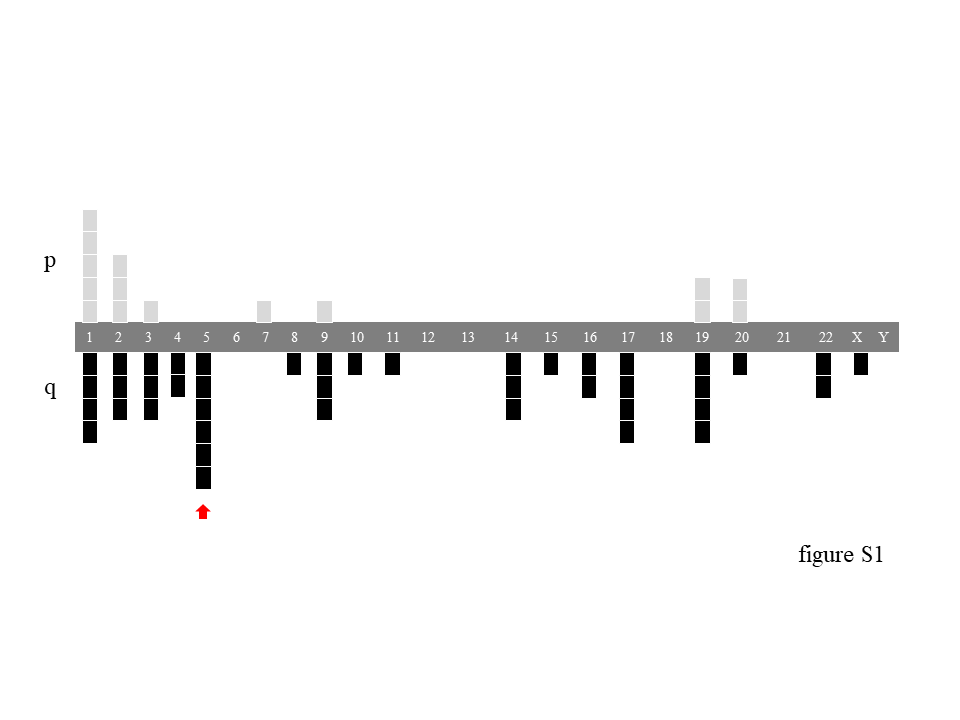


**S1 Fig.** Mapping of 57 candidate genes identification of pull-down assay. ‘p’ refers to the short arm of each chromosome, and ‘q’ refers to the long arm. Numbers indicate chromosome number. Red arrow shows 6 genes coding on 5q.
